# Peripheral brain-derived neurotrophic factor is related to cardiovascular risk factors in active and inactive elderly men

**DOI:** 10.1590/1414-431X20165253

**Published:** 2016-06-20

**Authors:** A. Zembron-Lacny, W. Dziubek, M. Rynkiewicz, B. Morawin, M. Woźniewski

**Affiliations:** 1 Department of Applied and Clinical Physiology, Faculty of Medicine and Health Sciences, University of Zielona Gora, Zielona Gora, Poland; 2 Department of Physiotherapy in Internal Diseases, Faculty of Physiotherapy, University School of Physical Education in Wroclaw, Wroclaw, Poland; 3 Department of Biological Basis of Sport, Faculty of Medicine and Health Sciences, University of Zielona Gora, Zielona Gora, Poland

**Keywords:** Neurotrophins, Lipoproteins, Oxidative damage, Aging, Cardiorespiratory fitness

## Abstract

Regular exercise plays an important preventive and therapeutic role in heart and vascular diseases, and beneficially affects brain function. In blood, the effects of exercise appear to be very complex and could include protection of vascular endothelial cells via neurotrophic factors and decreased oxidative stress. The purpose of this study was to identify the age-related changes in peripheral brain-derived neurotrophic factor (BDNF) and its relationship to oxidative damage and conventional cardiovascular disease (CVD) biomarkers, such as atherogenic index, C-reactive protein (hsCRP) and oxidized LDL (oxLDL), in active and inactive men. Seventeen elderly males (61-80 years) and 17 young males (20-24 years) participated in this study. According to the 6-min Åstrand-Rhyming bike test, the subjects were classified into active and inactive groups. The young and elderly active men had a significantly better lipoprotein profile and antioxidant status, as well as reduced oxidative damage and inflammatory state. The active young and elderly men had significantly higher plasma BDNF levels compared to their inactive peers. BDNF was correlated with VO_2_max (r=0.765, P<0.001). In addition, we observed a significant inverse correlation of BDNF with atherogenic index (TC/HDL), hsCRP and oxLDL. The findings demonstrate that a high level of cardiorespiratory fitness reflected in VO_2_max was associated with a higher level of circulating BDNF, which in turn was related to common CVD risk factors and oxidative damage markers in young and elderly men.

## Introduction

Regular exercise is known to improve the physiological performance of skeletal and cardiac muscles and decrease the incidence of a wide range of diseases, including heart and vascular diseases, certain kinds of cancers, type 2 diabetes, etc. (1). In the last decade, it has become clear that regular exercise beneficially affects brain function as well, and could play an important preventive and therapeutic role in stroke and degenerative diseases (2,3). The effects of exercise appear to be very complex and could include enhanced angiogenesis, neurogenesis and mitogenesis via neurotrophic factors, and reduced oxidative stress (4).

Brain-derived neurotrophic factor (BDNF) is a part of the neurotrophic family of growth factors, and is responsible for enhancing progenitor cell proliferation and differentiation, cell growth and regeneration processes, neuronal survival, and long-term synaptic remodeling and plasticity. The effects of neurotrophins are mediated by a family of specific transmembrane tyrosine kinase receptors, of which, TrkB is the primary signal transduction receptor for BDNF (5,6). BDNF has been identified as a key component of the hypothalamic pathway that controls body mass and energy homeostasis (3). Kermani et al. (7) and Matthews et al. (8) have shown that BDNF appears to be a major player not only in central metabolic pathways but also as a regulator of angiogenesis and muscle regeneration where it is responsible for mobilization of hematopoietic progenitor cells as well as proliferation and differentiation of satellite cells.

Studies have demonstrated that physical exercise can increase peripheral BDNF levels. In humans, BDNF release from the brain was observed at rest and increased even 3-fold during exercise. Both at rest and during exercise, the brain contributed to 70-80% of the circulating BDNF (9). This suggests that the brain is a major, but not the sole, contributor to peripheral BDNF. However, Matthews et al. (8) demonstrated that muscle-derived BDNF did not appear to be released into circulation. Interestingly, BDNF synthesis increased by muscle contraction enhances fat oxidation in an AMPK-dependent fashion, most probably by acting in an autocrine and/or paracrine manner within skeletal muscle. Hence, BDNF has been identified as a novel contraction-induced protein that may contribute to the multiple health benefits associated with physical activity (3).

It has been demonstrated that factors, such as age, race, gender, and weight, have an influence on stored and circulating BDNF levels in humans (10,11). Coelho et al. (12) provided a review of six studies that examined the effect of exercise on BDNF in elderly individuals. They found that it was not possible to establish a recommendation protocol for the type and intensity of physical exercise required to produce an increase in BDNF levels. However, moderate-intensity exercises, such as walking, seem to be the most effective to promote an increase in peripheral levels of BDNF among the elderly (13). More recently, Huang et al. (14) reviewed 32 experimental studies and reported that peripheral BDNF concentrations were elevated by acute and chronic aerobic exercise. The majority of the studies demonstrated that strength training had no influence on peripheral BDNF.

There has been a growing body of research focusing on the relationship between physical activity and BDNF levels in peripheral blood. Three review articles in this area have been published. The first one presented a systematic review of experimental studies on the effects of exercise on peripheral BDNF in healthy subjects and persons with a disease or disability, including methodological issues (15). The results from a few experimental studies showed contrary relationships between the peripheral BDNF level and habitual physical activity or cardiorespiratory fitness (16-19). Therefore, the purpose of this study was to identify the age-related changes in peripheral BDNF and its relationship to oxidative damage and conventional health markers in active and inactive men.

## Material and Methods

Thirty elderly men were recruited from the University of the Third Age U3T (age 60-80 years) who did not declare *sport* experience ten years after retirement. The current health status and lifestyle of the subjects were recorded using the health history questionnaire (20,21). Exclusion criteria, based on the assessment of the responsible physician and investigator, were acute infectious diseases, cardiovascular, neurological and musculoskeletal disturbances as well as blood transfusion. Twenty-three left the study because of high dyslipidemia, hypertension, dementia and intake of some medications, e.g., antidepressants, beta-adrenergic blocking agents and nonsteroidal anti-inflammatory drugs. Seventeen healthy untrained males, ages 20-24 years, composed a reference group (Table 1).

**Table:**
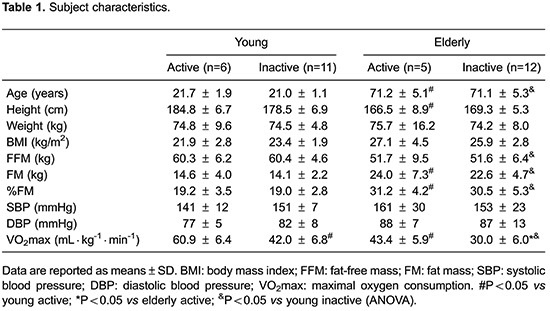

All subjects were informed of the aim of the study and gave their written consent for participation in the project. The Bioethics Commission at Medical University Poznan (N° 392/13), in accordance with the Helsinki Declaration, approved the protocol of the study.

### Body composition

Body mass and body composition (fat-free mass, FFM, and fat mass, FM) were estimated by a bioelectrical impedance method using Tanita body composition analyzer BC-418MA (Japan) calibrated prior to each test session in accordance with the manufacturer's guidelines. Duplicate measurements were taken with the participant in a standing position; the average value was used for the final analysis. The recurrence of measurement was 98%. The measurements were taken between 7:00 and 8:00 am, before blood sampling.

### Cardio-respiratory fitness

Maximal oxygen consumption (VO_2_max) was measured via the indirect method known as the Åstrand-Rhyming bike test (6-min submaximal exercise test) recommended for both men and women of various ages which relies on the linear relationship between heart rate and VO_2_ to predict VO_2_max (20). Each subject performed a 6-min submaximal exercise test using the ergometer bike Cardiac Rehab (Monark Ergomedic 818E, Germany). Initially, subjects rested for 15 min prior to the measurement of resting heart rate. Seat height and handlebars were adjusted to fit the subject prior to the test. According to normative data for submaximal exercise test, young and elderly men who reached the values of VO_2_max >52 mL·kg^-1^·min^-1^ and >5 mL·kg^-1^·min^-1^, respectively, were classified as active (high active level), and the remaining subjects as inactive (average and low active level).

### Blood sampling

Blood samples were taken from the elbow vein between 8:00 and 9:00 am using S-Monovette-EDTA tubes (Sarstedt, Austria). Within 20 min, they were centrifuged at 1000 *g* at 4°C for 10 min. Aliquots of plasma were stored at -80°C.

### Lipid profile and C-reactive protein

Plasma total cholesterol, high-density lipoproteins (HDL) and low-density lipoproteins (LDL) as well as triglycerides were determined by the professional laboratory company Diagnostyka (Poland, ISO 15189). C-reactive protein (hsCRP) concentration was determined in duplicate by DRG ELISA kit (USA). Detection limit was 0.001 mg/L, and coefficient of variation (CV) for the hsCRP kit was <3%.

### Framingham risk score

To calculate the subject's overall CVD risk, the conventional CVD risk factors that were measured were applied to the equations based on the Framingham study (22).

### Oxidative stress markers

Total plasma antioxidant status (TAS) and lipid peroxides (LPO) concentrations were determined in duplicate using Oxis Research kits (USA). TAS and LPO detection limits were 0.125 and 0.1 nmol/mL, respectively. Plasma protein carbonyls (PC) concentration, a marker of oxidative protein damage, was determined in duplicate using Alexis immune-assay kit (USA). PC detection limit was 0.1 nmol/mL. Oxidized low-density lipoprotein (oxLDL) was determined in duplicate using EIAab Science kit (China). oxLDL detection limit was 0.312 ng/mL. The intra-assay CV for all used kits was between 5 and 8%.

### Brain-derived neurotrophic factor

Plasma BDNF concentration was evaluated in duplicate by the R&D Systems ELISA kit (USA). BDNF detection limit was 20 pg/mL. The intra-assay CV for the BDNF kit was below 5%.

### Statistical analysis

Statistical analyses were performed using the statistical software Statistica 12 (StatSoft Inc., USA). All data were tested for distribution normality using the Shapiro-Wilk test. The values of W for BDNF, health and oxidative stress markers were close to one, therefore statistical significance was assessed using two-way analysis of variance (ANOVA) and *post hoc* tests (Tukey's HSD). Associations among measured parameters were analyzed using Pearson's linear regression (r coefficient). Statistical significance was set at P<0.05. Results are reported as means±SD.

## Results

The active and inactive subjects were successfully matched for age, BMI and body composition in both the young and elderly groups. The most significant differences concerning body composition were observed between the young group and the elderly inactive men. FM content was highly correlated with concentrations of hsCRP (r=0.709, P<0.001), oxLDL (r=0.497, P<0.01) and 10-year CVD risk (r=0.659, P<0.001). As expected, active subjects had significantly higher VO_2_max values (Table 1).

Both young and elderly active men had significantly better lipoprotein profile and reduced hsCRP levels compared to their inactive peers. hsCRP concentration in inactive elderly men was 5-fold higher compared to the young groups. This demonstrates that aging led to a chronic inflammatory state but regular physical activity can counteract this effect (Table 2).

**Table:**
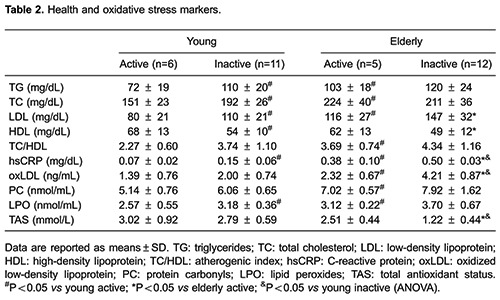

Multiple comparisons revealed that the elderly inactive men had significantly higher oxLDL and lower TAS compared to either the young (P<0.001) active or inactive (P<0.001) groups (Table 2). The percentage of 10-year CVD risk increased in inactive elderly men by 3% compared to the active elderly, and by 16% compared to the young groups. Similarly to the study by Björk et al. (23) and by Narotzki et al. (24), the 10-year CVD risk was significantly correlated with oxLDL level (r=0.738, P<0.001) and antioxidant status (r=-0.656, P<0.001).

Significant correlations were found between LDL and HDL, and oxidative damage markers such as oxLDL, PC and LPO, as well as antioxidant status (Table 3). TAS was 2-fold lower in inactive than active elderly men. All young men demonstrated sufficient antioxidant status, i.e., >1.3 mmol/L, whereas the inactive elderly group demonstrated borderline (<1.3 mmol/L) or low antioxidant status (<1 mmol/L). There was a positive correlation between VO_2_max and TAS (r=0.589, P<0.001). This clearly shows that aging and inactivity enhance oxidative stress which may relate to impairment of antioxidants and have a detrimental health potential.

**Table:**
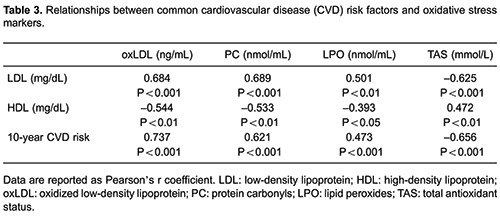

**Figure 1:**
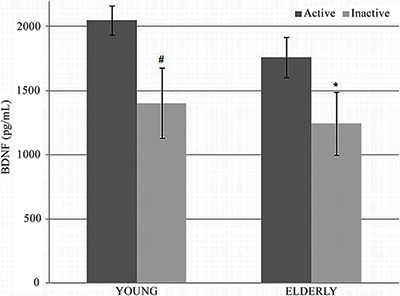
Main effect of age on plasma brain-derived neurotrophic factor (BDNF) levels between young (n=7) and elderly (n=17) men. ^#^P<0.05 *vs* young active; *P<0.05 *vs* elderly active (ANOVA).

**Figure 2:**
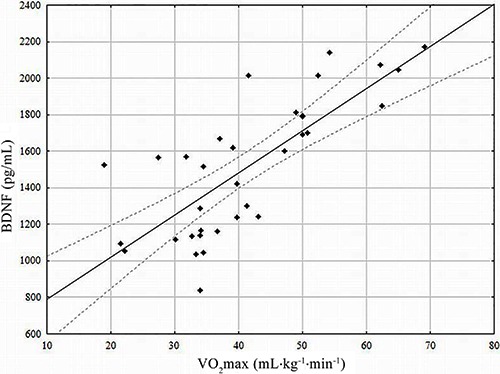
Correlation results between maximal oxygen consumption (VO_2max_) and brain-derived neurotrophic factor (BDNF) concentration. r=0.765, P<0.001 (Pearson's linear regression).

The young men had significantly (P<0.01) higher concentrations of BDNF (1630±391 pg/mL) than the elderly (1395±327 pg/mL). The active subjects, both young and elderly men, had significantly higher BDNF levels compared to their inactive peers (Figure 1). In elderly active men, BDNF concentration was 25% higher (P<0.05) than in inactive young men. BDNF concentration was directly correlated with VO_2_max level (Figure 2). In addition, we found an inverse relationship between BDNF and CVD risk factors, such as atherogenic index, hsCRP and oxLDL (Table 4).

**Table:**
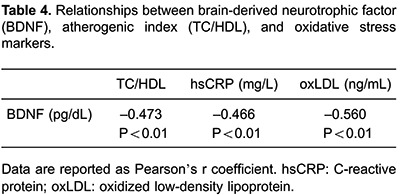

## Discussion

Aging is associated with immunosenescence and is accompanied by a chronic inflammatory state which contributes to a metabolic syndrome and cardiovascular consequences. CVD and endothelial dysfunction are characterized by a chronic alteration of inflammatory function. Markers of inflammation and the innate immune response including C-reactive protein and several other pro-inflammatory molecules are linked to the occurrence of myocardial infarction and stroke in healthy elderly populations (25).

HsCRP is an acute phase protein that is considered to be a general inflammatory marker. While the exact biological actions of hsCRP have not been established, its high levels predict risk of mobility/disability and are associated with accelerated decline in physical performance in older adults (26). The observed 5-fold increase in hsCRP concentration in elderly men indicates the presence of low-grade inflammation during aging. Inflammaging is considered a predictor of fragility and is currently accepted as a pathogenic factor in the development of several age-related diseases, such as CVD. Nevertheless, the precise etiology of inflammaging and its potential causal role in adverse health outcomes remain largely unknown. The identification of pathways that control age-related inflammation across multiple systems is, therefore, important in order to understand whether treatments that modulate inflammaging may be beneficial in the elderly population (27).

An increased FM content in elderly men was related to a systemic inflammation state partially promoted by pro-oxidative processes. The levels of oxLDL, PC and LPO were elevated in elderly men and highly correlated with common CVD risk factors such as LDL, HDL and Framingham score (Table 3). The relationships between oxidative stress markers and CVD risk factors suggest that, at the very least, plasma pro-oxidative molecules may serve as indicators of subclinical atherosclerosis development in clinically healthy men. OxLDL is produced by the oxidation of LDL, migrates into the subendothelial space, and promotes endothelial dysfunction, vascular remodeling, plaque rupture and thrombosis (28).

Many pathological changes associated with aging are explained by the irreversible accumulation of pro-oxidative molecules (29). Therefore, one would expect older indi-viduals to have an impairment in antioxidant defense. We observed significantly decreased plasma TAS in elderly men compared to the young subjects. Although acute exercise results in elevated oxidative stress, some recent studies have found that both low and high physical fitness levels help maintain better antioxidant potential in older adults (1,30). Our study demonstrated that active individuals had higher TAS as well as lower oxLDL, PC and LPO than their sedentary peers. This confirms previous results of reduced oxidative damage following an exercise intervention (23). Also, our positive correlation between VO_2_max and TAS further supports the hypothesis that elevated physical activity enhances antioxidant defense.

Low-grade inflammation and oxidative stress are common during aging and seem to be linked to degenerative diseases (25-27). Regular physical exercise can help to reduce an inflammatory state and to improve tissue function (1,3). BDNF is considered one of the most plausible factors involved in the health benefits associated with physical activity (3,4,14). In the present study, we found that high levels of cardio-respiratory fitness (estimated by the Åstrand-Rhyming test) was related with increased circulating BDNF concentration in both young and elderly men. Individuals with higher VO_2_max demonstrated elevated plasma BDNF level. The majority of previous studies suggest an inverse association between BDNF and habitual physical activity or cardiorespiratory fitness (16,31,32), which seems to be in conflict with the results from experimental studies (18,19,33,34). According to Huang et al. (14), discrepancy in results may be caused by various sample sizes, ranging from 8 to 955, and different measures of physical activity such as VO_2_max, lifestyle questionnaire and energy expenditure. Therefore, it is difficult to observe the unambiguous relationship between BDNF and physical activity due to limitations, such as selected study populations, subjective measures of physical activity, and uncontrolled confounding factors (14).

The biological mechanisms underlying the relationship between peripheral BDNF and habitual physical activity is still unclear, but several potential explanations are suggested. Firstly, the brain is a major source for increased plasma BDNF in physically active individuals (9). Warnn et al. (35) reported that the FNDC5 protein is released during exercise from skeletal muscles, inducing BDNF from hippocampus. Secondly, most of the peripheral BDNF is stored in platelets which might release BDNF in response to exercise at the site of injury to promote vascular repair (36). Furthermore, immune cells also release BDNF, which could sustain tissue repair in inflamed places (37). According to a recent study, BDNF may have a protective role in counteracting the inflammatory effects of hsCRP (11).

This is the first study to demonstrate that high plasma BDNF levels associated with physical activity may have an impact on CVD risk factors such as atherogenic index, oxLDL and hsCRP (Table 4). Recently, Noren Hooten et al. (11) observed that BDNF can reduce hsCRP levels and inhibit CRP-induced oxidative DNA damage in African-American women. Currently, BDNF is identified as a novel contraction-induced protein that may contribute to the multiple health benefits associated with physical exercise. In contrast, inactivity and low circulating BDNF levels are associated with many chronic disorders such as obesity, type 2 diabetes, cardiovascular diseases, depression, dementia and cognitive impairment (3). Matthews et al. (8) even suggested the possibility of using BDNF analogues as a possible therapy to treat metabolic diseases.

Our findings demonstrate that high levels of cardiorespiratory fitness reflected in VO_2_max were associated with higher levels of circulating BDNF, which in turn were related to common CVD risk factors and oxidative damage markers in young and elderly men. However, it is too early to draw a clear conclusion on a clinically relevant relationship between BDNF and habitual physical activity due to the small sample size.

## References

[1] Radak Z, Chung HY, Goto S (2005). Exercise and hormesis: oxidative stress-related adaptation for
successful aging. Biogerontology.

[2] Mattson MP, Wan R (2005). Beneficial effects of intermittent fasting and caloric restriction on
the cardiovascular and cerebrovascular systems. J Nutr Biochem.

[3] Pedersen BK, Pedersen M, Krabbe KS, Bruunsgaard H, Matthews VB, Febbraio MA (2009). Role of exercise-induced brain-derived neurotrophic factor production
in the regulation of energy homeostasis in mammals. Exp Physiol.

[4] Adlard PA, Cotman CW (2004). Voluntary exercise protects against stress-induced decreases in
brain-derived neurotrophic factor protein expression. Neuroscience.

[5] Hunsberger J, Austin DR, Henter ID, Chen G (2009). The neurotrophic and neuroprotective effects of psychotropic
agents. Dialogues Clin Neurosci.

[6] Seifert T, Brassard P, Wissenberg M, Rasmussen P, Nordby P, Stallknecht B (2010). Endurance training enhances BDNF release from the human
brain. Am J Physiol Regul Integr Comp Physiol.

[7] Kermani P, Rafii D, Jin DK, Whitlock P, Schaffer W, Chiang A (2005). Neurotrophins promote revascularization by local recruitment of TrkB+
endothelial cells and systemic mobilization of hematopoietic
progenitors. J Clin Invest.

[8] Matthews VB, Astrom MB, Chan MH, Bruce CR, Krabbe KS, Prelovsek O (2009). Brain-derived neurotrophic factor is produced by skeletal muscle cells
in response to contraction and enhances fat oxidation via activation of
AMP-activated protein kinase. Diabetologia.

[9] Rasmussen P, Brassard P, Adser H, Pedersen MV, Leick L, Hart E (2009). Evidence for a release of brain-derived neurotrophic factor from the
brain during exercise. Exp Physiol.

[10] Lommatzsch M, Zingler D, Schuhbaeck K, Schloetcke K, Zingler C, Schuff-Werner P (2005). The impact of age, weight and gender on BDNF levels in human platelets
and plasma. Neurobiol Aging.

[11] Noren Hooten N, Ejiogu N, Zonderman AB, Evans MK (2015). Protective effects of BDNF against C-reactive protein-induced
inflammation in women. Mediators Inflamm.

[12] Coelho FG, Gobbi S, Andreatto CA, Corazza DI, Pedroso RV, Santos-Galduroz RF (2013). Physical exercise modulates peripheral levels of brain-derived
neurotrophic factor (BDNF): a systematic review of experimental studies in the
elderly. Arch Gerontol Geriatr.

[13] Leckie RL, Oberlin LE, Voss MW, Prakash RS, Szabo-Reed A, Chaddock-Heyman L (2014). BDNF mediates improvements in executive function following a 1-year
exercise intervention. Front Hum Neurosci.

[14] Huang T, Larsen KT, Ried-Larsen M, Moller NC, Andersen LB (2014). The effects of physical activity and exercise on brain-derived
neurotrophic factor in healthy humans: A review. Scand J Med Sci Sports.

[15] Knaepen K, Goekint M, Heyman EM, Meeusen R (2010). Neuroplasticity - exercise-induced response of peripheral
brain-derived neurotrophic factor: a systematic review of experimental studies in
human subjects. Sports Med.

[16] Currie J, Ramsbottom R, Ludlow H, Nevill A, Gilder M (2009). Cardio-respiratory fitness, habitual physical activity and serum brain
derived neurotrophic factor (BDNF) in men and women. Neurosci Lett.

[B17] Babaei P, Damirchi A, Mehdipoor M, Tehrani BS (2014). Long term habitual exercise is associated with lower resting level of
serum BDNF. Neurosci Lett.

[B18] Whiteman AS, Young DE, He X, Chen TC, Wagenaar RC, Stern CE (2014). Interaction between serum BDNF and aerobic fitness predicts
recognition memory in healthy young adults. Behav Brain Res.

[19] Erickson KI, Voss MW, Prakash RS, Basak C, Szabo A, Chaddock L (2011). Exercise training increases size of hippocampus and improves
memory. Proc Natl Acad Sci U S A.

[20] Astrand PO (1956). Human physical fitness with special reference to sex and
age. Physiol Rev.

[21] Durstine JL, Moore GE (2003). ACSM's exercise management for persons with chronic diseases and
disabilities.

[22] Wilson PW, D'Agostino RB, Levy D, Belanger AM, Silbershatz H, Kannel WB (1998). Prediction of coronary heart disease using risk factor
categories. Circulation.

[23] Björk L, Jenkins NT, Witkowski S, Hagberg JM (2012). Nitro-oxidative stress biomarkers in active and inactive
men. Int J Sports Med.

[24] Narotzki B, Reznick AZ, Mitki T, Aizenbud D, Levy Y (2014). Enhanced cardiovascular risk and altered oxidative status in elders
with moderate excessive body fat. Rejuvenation Res.

[25] Guarner V, Rubio-Ruiz ME (2015). Low-grade systemic inflammation connects aging, metabolic syndrome and
cardiovascular disease. Interdiscip Top Gerontol.

[B26] Verghese J, Holtzer R, Lipton RB, Wang C (2012). High-sensitivity C-reactive protein and mobility disability in older
adults. Age Ageing.

[27] Franceschi C, Campisi J (2014). Chronic inflammation (inflammaging) and its potential contribution to
age-associated diseases. J Gerontol A Biol Sci Med Sci.

[28] Stocker R, Keaney JF (2004). Role of oxidative modifications in atherosclerosis. Physiol Rev.

[29] Sohal RS, Weindruch R (1996). Oxidative stress, caloric restriction, and aging. Science.

[30] Ceci R, Beltran Valls MR, Duranti G, Dimauro I, Quaranta F, Pittaluga M (2014). Oxidative stress responses to a graded maximal exercise test in older
adults following explosive-type resistance training. Redox Biol.

[31] Joung SH, Kim J, Davis JM, Blair SN, Cho HC (2011). Association among basal serum BDNF, cardiorespiratory fitness and
cardiovascular disease risk factors in untrained healthy Korean
men. Eur J Appl Physio.

[32] Nofuji Y, Suwa M, Moriyama Y, Nakano H, Ichimiya A, Nishichi R (2008). Decreased serum brain-derived neurotrophic factor in trained
men. Neurosci Lett.

[33] Zoladz JA, Pilc A, Majerczak J, Grandys M, Zapart-Bukowska J, Duda K (2008). Endurance training increases plasma brain-derived neurotrophic factor
concentration in young healthy men. J Physiol Pharmacol.

[34] Correia PR, Scorza FA, Gomes da SS, Pansani A, Toscano-Silva M, de Almeida AC (2011). Increased basal plasma brain-derived neurotrophic factor levels in
sprint runners. Neurosci Bull.

[35] Warnn CD, White JP (2013). FNDC5 a muscle protein induces BDNF release from
hippocampus. Cell Metab.

[36] Yamamoto H, Gurnay ME (1990). Human platelets contain brain-derived neurotrophic
factor. J Neurosci.

[37] Colombo E, Bedogni F, Lorenzetti I, Landsberger N, Previtali SC, Farina C (2013). Autocrine and immune cell-derived BDNF in human skeletal muscle:
implications for myogenesis and tissue regeneration. J Pathol.

